# PAN2HGENE–tool for comparative analysis and identifying new gene products

**DOI:** 10.1371/journal.pone.0252414

**Published:** 2021-05-28

**Authors:** Mônica Silva de Oliveira, Jorianne Thyeska Castro Alves, Pablo Henrique Caracciolo Gomes de Sá, Adonney Allan de Oliveira Veras

**Affiliations:** 1 Postgraduate Program in Applied Computing, Federal University of Pará Campus Tucuruí (CAMTUC-UFPA), Pará, Brazil; 2 Pará State University Campus Marabá (UEPA), Pará, Brazil; 3 Federal Rural University of Amazonia Campus Tomé-Açu (UFRA), Pará, Brazil; University of Oxford, UNITED KINGDOM

## Abstract

Advances in next-generation sequencing (NGS) platforms have had a positive impact on biological research, leading to the development of numerous omics approaches, including genomics, transcriptomics, metagenomics, and pangenomics. These analyses provide insights into the gene contents of various organisms. However, to understand the evolutionary processes of these genes, comparative analysis, which is an important tool for annotation, is required. Using comparative analysis, it is possible to infer the functions of gene contents and identify orthologs and paralogous genes via their homology. Although several comparative analysis tools currently exist, most of them are limited to complete genomes. PAN2HGENE, a computational tool that allows identification of gene products missing from the original genome sequence, with automated comparative analysis for both complete and draft genomes, can be used to address this limitation. In this study, PAN2HGENE was used to identify new products, resulting in altering the alpha value behavior in the pangenome without altering the original genomic sequence. Our findings indicate that this tool represents an efficient alternative for comparative analysis, with a simple and intuitive graphical interface. The PAN2HGENE have been uploaded to SourceForge and are available via: https://sourceforge.net/projects/pan2hgene-software

## Introduction

Next-generation sequencing (NGS) platforms have sparked a dramatic change in the history of genome sequencing processes. NGS facilitates complete sequencing of genomes at relatively low costs, allowing the development of several other analyses including comparative analysis [1, 2].

The main advantages of NGS are the production of large amounts of data, lower costs, and reduced time to sequencing. However, the emergence of these tools led to an additional challenge of handling large volumes of data, affirming the need to develop efficient ways for storage and management of data [1]. Over the years, bioinformatics approaches have contributed to improved handling and storage of data. Public databases, including NCBI, EBI, and DDBJ, are used by researchers from various fields for handling such data. These databases store diverse biological information including sequencing, annotation, and genome assembly data [3].

Although these platforms provide understanding regarding the genomics of an organism, new tools are required to gain insights into the functions of gene contents. Comparative analysis is effective to this goal, with high accuracy in terms of the structural annotation process, as this type of analysis allows identification of orthologs genes by homology [3, 4].

Numerous computational tools have been developed to perform this type of analysis. Among these, the ABC software was created for interactive navigation of genomic data and can be used to perform multiple sequence alignments. This tool allows quantitative data on the alignments and annotations of the genes under study to be displayed simultaneously, thus highlighting the similarities in their sequences and evolutionary rates. Its purpose is to facilitate comparative sequence analyses, such as visualization of phylogenetic trees and generation of summary graphs [5].

PanTools is a software package that features genome annotation, sequence addition, gene cluster, genome reconstruction, pan-genome comparison, and query functionality. Its implementation is based on the Neo4j graph database, demanding the application of large sets of eukaryotic genomes (62 Escherichia coli genomes, 93 yeast genomes, and 19 Arabidopsis thaliana genomes). This program facilitates the construction of pan-genomic databases of many genomes with extensions for sequence addition and ontology annotations, among others. According to its creators, PanTools is the starting point for a collection base and is used as a linear reference in the field of comparative genomics [6].

ITEP, an integrated toolkit for genome exploration, consists of a series of command scripts that allow identification, comparison, and curation analysis of protein families. This tool uses a set of Python libraries to access genome information data and executes via scripts, workflows, and analyses related to a complete collection of genomes. ITEP proves to be an advantageous and flexible option for comparative analysis of microbial pan-genomes as it has been designed in modules, and thus allows the addition of functionalities and workflows for analysis [7].

Fast-D is a local annotation tool that allows the assignment of orthologs based on a reference genome. Using fasta files as input, this tool allows users to customize parameters and reference databases, offering command-line options and editing of the original configuration file. Fast-D has two annotation phases—structural and functional. The structural phase predicts biological characteristics (CDSs, RNAz, and CRISPRs), and the functional phase provides the functions of proteins predicted in CDSs. Each step of the annotation process is implemented through modules developed in Python, allowing the addition of extensions and new features. Fast-D provides better results for well-characterized organisms (Actinobacteria, Firmicutes, and Proteobacteria) than for less-studied species. It is possible to present numerous uncharacterized genes in this standard database [8].

Although the tools presented above make important contributions to facilitating comparative analyses, most of these tools have limitations when running on the web interface or have extensive command lines which lead to an increase in the user’s difficulty of use. Thus, we present PAN2HGENE, a computational tool that allows the identification of gene products missing from the original genomic sequence and performs automated comparative analysis using both complete and draft genomes, through a simple and intuitive graphical interface.

## Materials and methods

### Tool validation

For tool validation, reads of fifteen *Escherichia coli* strains were used. These data are available at the NCBI database in SRA format (https://www.ncbi.nlm.nih.gov/sra) and were downloaded using fastq-dump, a script from the SRA toolkit package (https://www.ncbi.nlm.nih.gov/sra/docs/sradownload/), using the—split-files <sra number> parameter for paired data. Table 1 lists the strains used, their SRAs, and library type.

**Table 1:** List of data used to validate the PAN2HGENE tool.

Organism	SRA Access Number	Type
*Escherichia coli* SE11	DRR015123	Single
*Escherichia coli* 042	ERR007646	Paired
*Escherichia coli* strain 4A	ERR2348864	Paired
*Escherichia coli* BL21(DE3)	ERR2596694	Paired
*Escherichia coli* 536	ERR351257	Paired
*Escherichia coli* O26:H11 str. 11368	ERR351259	Paired
*Escherichia coli* O103:H2 str. 12009	ERR351260	Paired
*Escherichia coli* KLY	SRR1424625	Paired
*Escherichia coli* P12b	SRR2000272	Paired
*Escherichia coli* strain AR_0061	SRR5168216	Paired
*Escherichia coli* strain 266917	SRR5470155	Paired
*Escherichia coli* strain ST540	SRR6111817	Paired
*Escherichia coli* PCN033	SRR8735180	Paired
*Escherichia coli* strain U13A	SRR8542038	Paired
*Escherichia coli* strain USML2	SRR8883688	Paired

In this analysis was used twelve complete genomes and three draft genomes. To evaluate the effectiveness of the tool, the data were analyzed using other comparative analysis tools, including PGAPWEB [9], PGAP [10], and PANWEB [11]. The criteria for choosing these tools was based on the fact that they all use PGAP as a tool to perform comparative analysis within their pipelines.

### Programming language and database

PAN2HGENE was developed using Java, a robust and multiplatform programming language, and NetBeans IDE 12.0 (https://www.oracle.com). The Swing library was used to create a graphical interface. The database manager used was MySQL 8.0.23. The following processes were carried out in addition to development.

### Mapping

Bowtie2 software version 2.3.5.1 (http://bowtie-bio.sourceforge.net/bowtie2/) was used to perform mapping of the raw reads against the input file, which can be draft genome or complete genome in FASTA format. As a result, a FASTQ file containing unmapped reads was generated [12].

### *De novo* assembly

The SPades software version 3.14.1 was used to assemble the dataset with unmapped reads, with default parameter values [13]. The files with raw paired reads are previously checked with the bbmap tool (sourceforge.net/projects/bbmap/) to address the existence of orphaned reads, called singletons. This treatment is necessary to avoid possible errors in the assembly process with Spades.

### Annotation

The comparative analysis needs the standardization of genomic sequence (complete genome or draft genome) of all organisms into the analysis. The standardization of the annotation performed in PAN2HGENE allows the user to choose between the web RAST platform (RAST) and Prokka—rapid prokaryotic genome annotation [14].

### Similarity

Blast software version 2 (https://blast.ncbi.nlm.nih.gov/Blast.cgi) was used to similarity search between the products from input file annotation against products obtained by the annotation process of dataset assembled of the unmapped reads.

### Comparative analysis

The PGAP 1.2.1 software [10] was used to perform the comparative analysis. All parameter values can be adjusted by the user on the PAN2HGENE graphical interface. The values of the parameters used in this study were as follows: method, GF; e value, 1 e-10; coverage and identity, 0.7. For analysis and visualization of the results, R software was used (https://www.r-project.org/).

### Pipeline

**Fig 1:**
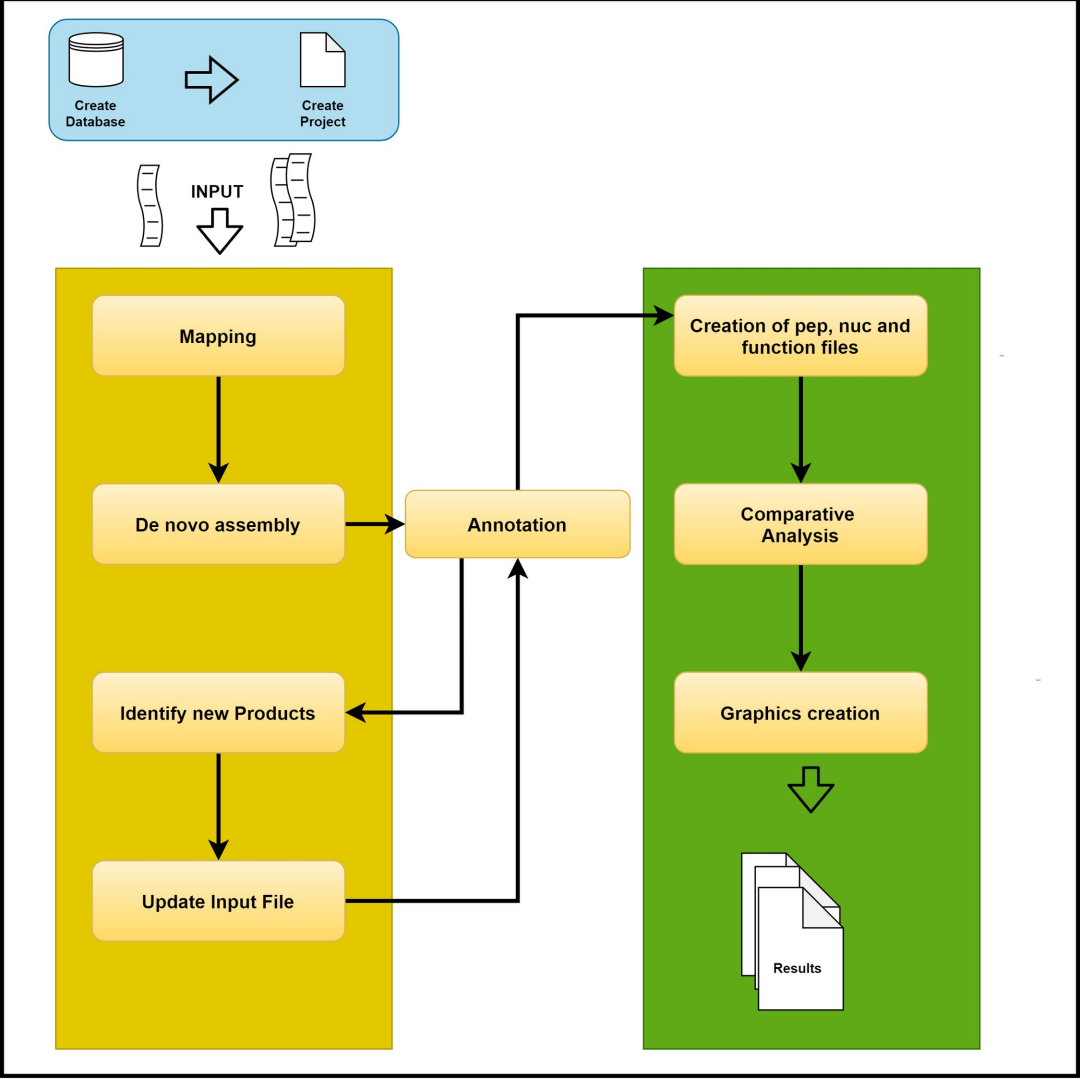
PAN2HGENE pipeline. Identification of gene products missing from the original sequence is shown in yellow and the process of comparative analysis is shown in green.

The PAN2HGENE pipeline is executed in two parts (Fig 1). The first consists of the process of identifying gene products missing the original genomic sequence. The second, on the other hand, is possible to carry out a comparative analysis of the target organisms of the study with their updated genomic sequences.

The steps that make up the first stage are: (i) input data: draft genome or complete genome sequence in the FASTA format (contigs or complete genome) used as reference and raw data (reads) in the FASTQ format; (ii) mapping: performed using Bowtie2, the raw data was mapped against the reference input file (FASTA) to obtain a FASTQ with unmapped reads; (iii) *de novo assembly* of unmapped reads using default parameter value; (iv) annotation of the reference input file and the result file generated from the *de novo assembly* of unmapped reads (both in FASTA).

The user can choose between the web RAST platform or Prokka. If the user chooses the annotation using RAST, at the end of this process, the annotation file is downloaded in EMBL format. However, if the user chooses to use Prokka, the annotation process occurs locally and the GBK annotation file is generated.

Identify new products (v): The CDS were extracted from annotation file and organized into a local database, the identification of new products was performed using Blast 2, the CDS extracted from the annotation file (reference input file) were mapped against CDS extracted from *de novo* assembly result; (vi) update input file: products that have not found any similarity in the BLAST analysis are added at the end of the input file. After the update process, the second round of annotation is performed.

The second part consists of: (i) file generation to comparative analysis: creation pep, nuc, and function files from annotation file (EMBL or GBK); (ii) comparative analysis using PGAP; and (iii) plotting of graphs results using R software.

## Results and discussion

### New product identification

PAN2HGENE identified missing products in most strains of *E*. *coli* analyzed. Table 2 shows the quantity of these new products, with fourteen of the fifteen strains analyzed presenting new products. The results are organized according to the Sequence Read Archive (SRA) number, new products, hypothetical protein quantity, average product size, and the total amount of the product.

**Table 2:** List of products identified through the PAN2HGENE pipeline.

SRA Number	New Products	Amount of hypothetical protein	Total Identified Products	Average Product Size
ERR007646	12	32	44	172pb
ERR2348864	480	369	849	596pb
ERR2596694	25	238	263	253pb
ERR351257	16	20	36	299pb
ERR351259	101	100	201	521pb
ERR351260	316	159	475	320pb
SRR1424625	03	10	13	241pb
SRR2000272	11	15	26	194pb
SRR5168216	98	84	182	523pb
SRR5470155	294	183	477	598pb
SRR6111817	265	204	469	579pb
SRR8542038	229	130	359	495pb
SRR8735180	2940	1977	4917	470pb
SRR8883688	865	457	1322	584pb

### Comparative analysis

Attempts to perform the analysis with the PANWEB software resulted in errors in the organisms with the following SRA numbers SRR5168216, SRR5470155, and SRR2000272, which made it impossible to use this tool in the process of comparing the results.

To perform PGAPweb tests, it was necessary to standardize the input files. According to the PGAPweb tool manual, input files can have three patterns (http://pgaweb.vlcc.cn/pgaweb.vlcc.cndoc). The standard chosen in this analysis was the generation of files with pep, nuc, and function extension, an ad hoc script was used to create these files.

The results of tests with PGAPweb indicated that, despite the generation of the pangenome graph, this tool did not provide the file for graph generation and the alpha values necessary for characterization of the pangenome graph to determine whether the pangenome was open or closed.

**Fig 2:**
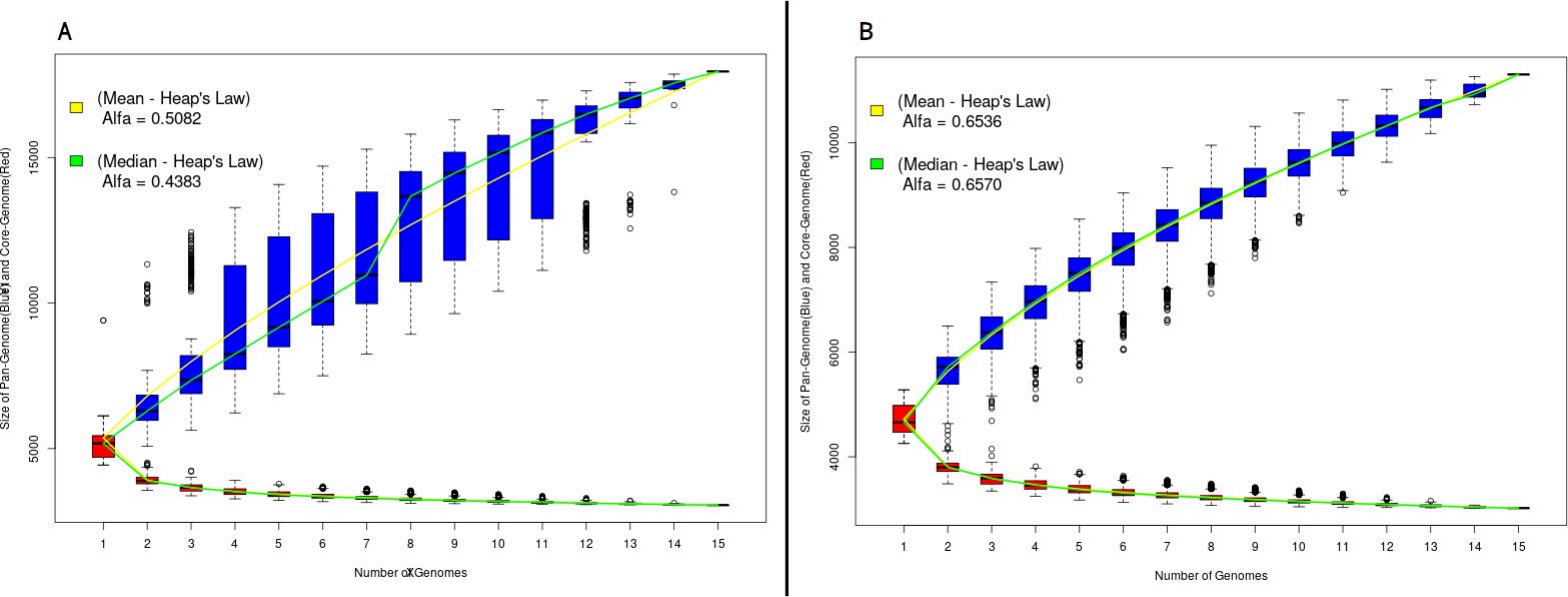
Pangenomic analysis. (A) Pangenome obtained using the PAN2HGENE software. (B) PGAP desktop results. The respective alpha values for the mean and median follow Heap’s law.

Based on this, the Desktop version of the PGAP software was used to perform the comparative analysis, using as input the files previously generated in the PGAPWeb test. The results obtained using PAN2HGENE and PGAP are shown in Fig 2. The first result refers to the pangenome analysis.

Comparison of the pangenomic analyses of the fifteen *E*. *coli* strains corroborates the results obtained in Gordienko’s study [15], which characterizes the pangenome as open. The alpha values obtained by analyzing the results were 0.4383 and 0.6570 for PAN2HGENE and PGAP, respectively. However, the products identified in the first part of the PAN2HGENE pipeline promoted a change in the calculated alpha value to the median without changing the number of organisms considered for analysis. Moreover, the outliers in the data sets were smaller in numbers and farther away as compared to other tools.

**Fig 3:**
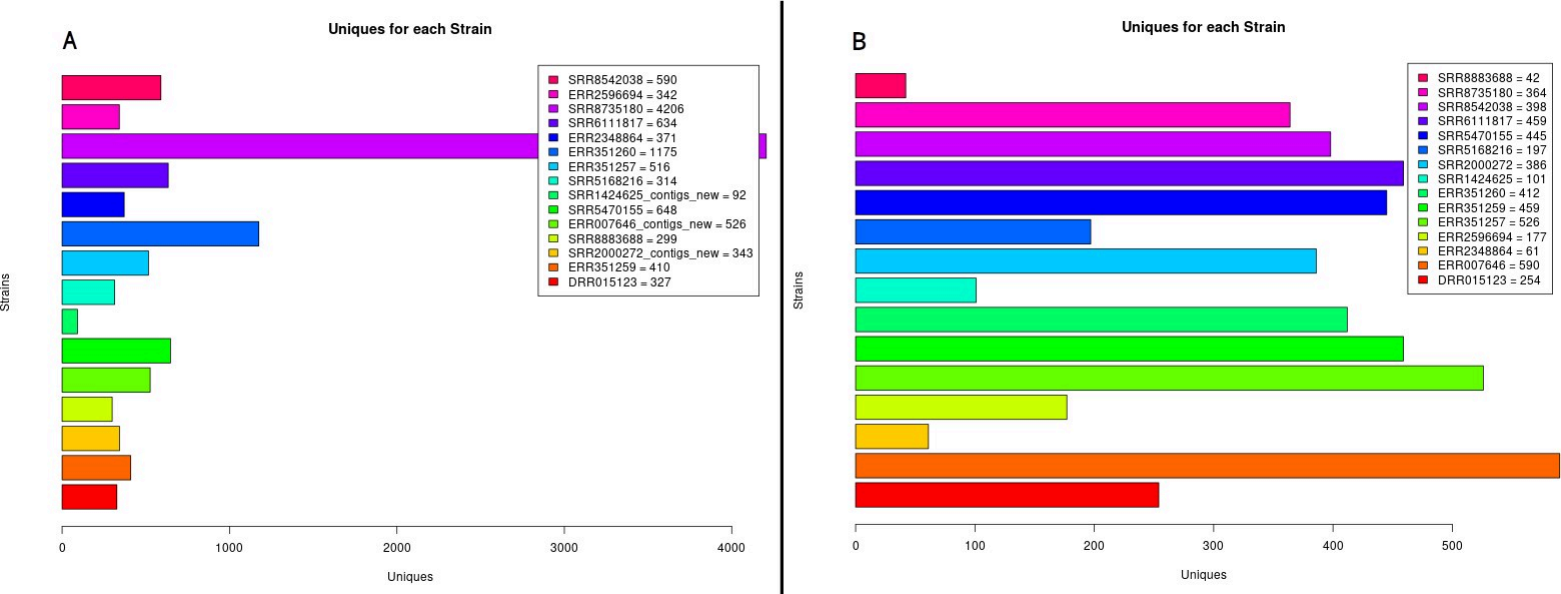
Unique genes for each strain. Graphs representing the number of unique genes identified in each species using PAN2HGENE (A) and PGAP (B) analyses.

**Fig 4:**
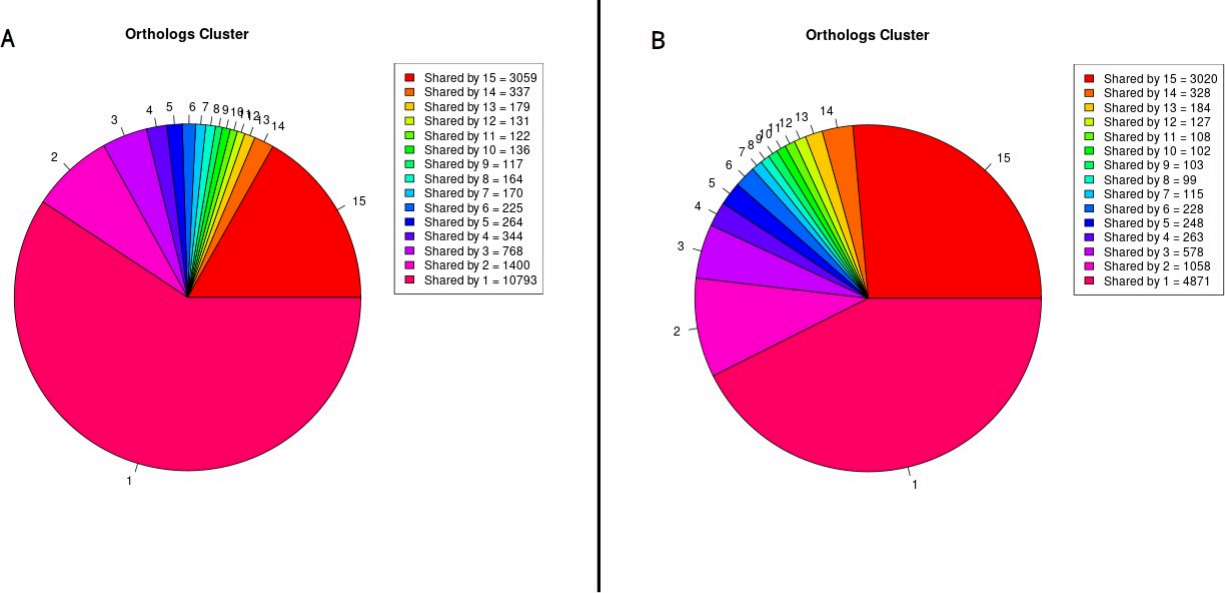
Orthologs genes. Pie chart representing the genes shared between strains using PAN2HGENE (A) and PGAP (B) analyses.

The impact of the addition of new products to analysis can be seen in the number of unique genes present in each organism. The results of PAN2HGENE showed that six (SRR8735180, ERR351260, SRR8542038, ERR2348864, SRR5470155, and SRR8883688) of the fifteen organisms analyzed presented a larger number of unique genes, as shown in Fig 3. Similarly, Fig 4 shows an increase in the number of genes present in the central genome.

**Fig 5:**
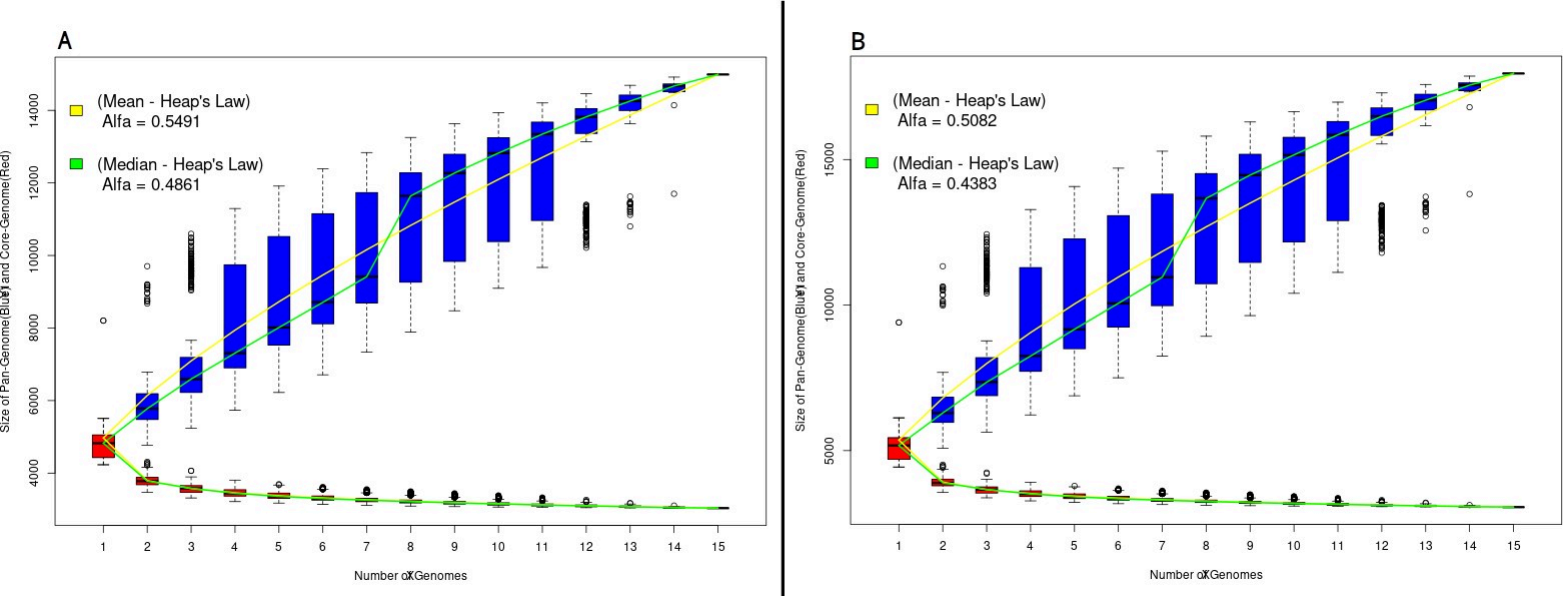
Result of pangenomic analysis using different annotation software. (A) pangenomic analysis using Prokka software and (B) analysis using the RAST platform.

A comparison was also made between the results of pangenomic analysis using the RAST and Prokka annotation software (Fig 5). Each annotation software performs its task following its strategy, it was observed that the comparative analysis can be influenced according to the annotation software used in the annotation standardization process. However, the graphical analysis of the results, as well as the analysis of the mean and median values of alpha demonstrate that the difference is not significant, but it does exist.

However, the analysis carried out using the PAN2HGENE software pipeline has as the main focus to maximize the representation of the genetic content of the organisms used in the analysis, resulting in a more accurate comparative analysis.

Table 3 highlights some functions performed by PAN2HGENE in comparison to the BPGA [16] and Roary [17] software that performs the comparative analysis.

**Table 3:** Summary about features performed by software BPGA, Roary and PAN2HGENE.

Features description	BPGA	Roary	PAN2HGENE
Database for processing step control.	-	-	OK
Execution to the command line.	OK	OK	OK
Plotting graphs about the results.	OK	OK—through external tools	OK
It has an intuitive graphical interface.	-	-	OK
Graphical results that are easy to analyze.	-	-	OK
Standardization of annotation in two different annotation systems.	-	-	OK
Maximizes the representation of the gene content of the organisms present in the analysis.	-	-	OK
It is possible to resume processing from the stop point in the event of failures.	-	-	OK

## Conclusion

Among the results produced with the execution of the pipeline, there are the figures on phylogenetic tree analysis: PanBased.Neighbor-joining.png, PanBased.UPGMA.png, SNPBased.ML.png, SNPBased.Neighbor-joining.png, SNPBased.UPGMA.png. Also, the barplot_uniques.png with unique genes for each strain, barplot.png containing the amount of orthologs genes shared by each strain, and boxplot.png which displays, simply and directly, the information about the pangenome and the core genome and the mean and median values of alpha, which directly assists the researcher in determining whether the pangenome is open or closed.

In addition to the figures, files are available in PDF format containing the gene products identified in the first stage of the pipeline, as well as all annotation files and other results from the execution of the PGAP software.

The results show that the PAN2HGENE software is an efficient alternative to perform comparative analysis. The software has a simple graphical interface and is intuitive, in case of possible failures (energy or internet) it is possible to resume processing from the point where it stopped, the status of each step is saved in a PAN2HGENE database. Identifying products that are missing from the original genome sequence provides a means of improving future analyzes.

As future work to be implemented in the next versions of this tool, there is the development of an XML parser that provides the user with the use of other engines to carry out the comparative analysis process, such as, BPGA, Roary.
